# Introduction to special issue *mind the gap between research and practice in the area of teachers’ support of metacognition and SRL*

**DOI:** 10.1007/s11409-021-09285-5

**Published:** 2021-11-08

**Authors:** Charlotte Dignath, Zemira Mevarech

**Affiliations:** 1 DIPF Leibniz Institute of Education Research, Frankfurt, Germany; 2 School of Education, Bar Ilan University, Ramat Gan, Israel

Since spring 2020, governments all over the world have temporarily and repeatedly closed schools in response to the COVID-19 pandemic. Learning at home requires a large amount of self-regulated learning (SRL) where students have to acquire and practice learning contents and skills independently without being provided with face to face support by their teacher. Current research reports that the main difficulties that students *and* teachers experience in these learning situations are related to SRL (Fischer et al., 2020).

But also in everyday learning situations at school, SRL has been found to contribute significantly to students’ learning progress, their motivation, and their achievement (Zimmerman & Bandura, 1994; Dent & Koenka, 2016). Acquiring skills for SRL is a necessity for students to become lifelong learners (Taranto & Buchanan, 2020), which has now been shown even more. However, when schools moved to distance learning, it not only became apparent how important SRL is, but also how poorly most students were prepared for SRL. This is even more the case for young students at primary school (Alvi & Gillies, 2021).

## The gap between research and practice regarding teachers’ promotion of SRL

Throughout the last two decades, researchers have advocated to promote SRL in the classroom. Whereas most researchers and policy makers agree with the significance of SRL for student learning, the challenges of implementing SRL in the classroom are many. One set of challenges includes teacher characteristics that affect their SRL practice in the classroom, such as their knowledge and beliefs about promoting SRL. Which prerequisites are necessary for teachers to successfully support SRL in their classroom? Another set of challenges contains issues in terms of how to support SRL and which components of SRL to address. Which type of instruction is required for an effective promotion of SRL? A third set of challenges regards teachers’ own learning: How can teachers and student-teachers be supported most optimally in fostering their students’ SRL? While all these challenges are related to the teachers, many questions are still open with regard to students’ SRL. For example: At what age are students ‘ready’ to implement SRL? Who are the students who benefit from SRL scaffolding? And to what extent does SRL scaffolding in one domain transfer to SRL implementation in other domains?

We have learned from research that teachers

- typically provide only little explicit support for SRL (Dignath & Veenman, 2021).
- perceive SRL to be desirable for their students (Dignath-van Ewijk & Van der Werf, 2012; Lombaerts et al., 2009), but not feasible to implement in their classrooms (De Smul et al., 2018).
- often hold misconceptions about which students benefit from SRL (Peeters et al., 2016; Zohar & Barzilai, 2015) and to what extent SRL scaffolding is needed for promoting lower vs. higher cognitive levels (Mevarech & Fan, 2018).

These results indicate a gap between the current state of knowledge about SRL in research and the teachers’ promotion of SRL in practice. What are the reasons for this gap, and how can SRL research contribute to close this gap?

## The contribution of this special issue to the field of SRL research

Our intend with this Special Issue is to provide an overview of current research investigating how teachers should learn to become more effective supporters of their students’ SRL. The goal is to provide research based data that would add to the understanding of teachers’ support of SRL in the classroom by highlighting the essence of interventions for teachers (pre-service and in-service teachers as well as novice and experienced teachers) and which specific aspects they should take into account in SRL scaffolding. The particular emphasis is on the effectiveness of teachers’ promotion of SRL, and how characteristics of the teacher and of the instruction moderate this effectiveness. In putting this special issue together, we invited authors who are tackling these issues in their own research. Each utilizes a different approach to the study of what makes teachers’ support of SRL particularly effective, including broadly varying theoretical perspectives, methodologies, and interventions.

This special issue includes four articles and two commentaries. The collection of contributions provides variety with respect to the sample (preservice and in-service teachers), research designs (e.g., path analyses, profile analyses, training studies), and the assessment of teachers’ promotion of SRL (e.g., teachers’ self-report, student-report, and classroom observations). All highlight the complexity of teachers’ support of SRL by carefully analyzing the effectiveness of different characteristics of teachers or their instruction on teachers’ SRL practice and on students’ learning progress.

In each article, the authors investigate a specific aspect of the teachers’ competencies (Vosniadou, Darmawan, Lawson, Van Deur, Jeffries, and Wyra: beliefs consistent and inconsistent with SRL theory; Dignath: beliefs, knowledge, and self-efficacy; Michalsky: professional vision focusing on teacher vs. focusing on students) or of the teachers’ instruction (Van Loon, Bayard, Steiner, and Roebers: child-directed vs. teacher-directed instruction) and relate these to students’ learning. While one article examines pre-service teachers in their dual role as learner and as future teacher (Vosniadou et al.), the other articles investigate pre-service or in-service teachers who are already teaching in the classroom. As an advancement to previous research, these studies all include additional student data to allow for assessing effects of teachers’ SRL practice on students’ learning and achievement.

In the opening article, Vosniadou, Darmawan, Lawson, Van Deur, Jeffries, and Wyra provide insights into associations between pre-service teachers’ beliefs about learning and teaching and their study practices and their academic performance. They added to existing SRL research by, for the first time, applying a conceptual change approach to the study of pre-service teachers’ beliefs about SRL. Whereas former research on teacher beliefs mainly regarded teacher beliefs about SRL in a linear way (e.g., Dignath, 2016; Lombaerts et al., 2009), Vosniadou and colleagues introduce a distinction between teacher beliefs consistent vs. inconsistent with SRL theory. Based on this approach, the authors conclude that SRL interventions for teachers can become more effective when taking into account (pre-service) teachers’ opposing beliefs about SRL as well in order to facilitate conceptual change.

The second article represents our own work with in-service teachers (Dignath). The new contribution of this article to the field of SRL research refers to the application of a generic model of teacher competence (Baumert & Kunter, 2013) to the context of teachers’ promotion of SRL. In the first study, we identified profiles of teacher competence regarding SRL which were based on teachers’ knowledge, beliefs, and self-efficacy about supporting SRL in the classroom. These profiles were associated with teachers’ self-reported SRL practice in the classroom. In our second study, we designed a professional development training that was evaluated on the teacher and the student level. The article reports findings with respect to the effectiveness of the training and insights into how the teachers’ competence profiles identified in study 1 moderate these training effects.

The third article, authored by Michalsky, examined how reflection prompts that focus on the teacher vs. on the students when watching a classroom video affected preservice teachers’ instruction of SRL strategies as well as their students’ achievement. The innovative aspect of her research program is to introduce the concept of professional vision (Goodwin 1994) into research about teachers’ support of SRL. The findings from her quasi-experimental study indicate in detail which type of reflection prompt is most beneficial for pre-service teachers’ learning about supporting their students’ SRL, and they illustrate the importance of teachers’ professional vision for their classroom practice and their students’ achievement.

The fourth article, by Van Loon, Bayard, Steiner, and Roebers, describes how teachers promote SRL and investigate associations between teachers’ instruction and students’ SRL. The most important novelty of their research is that it addresses the association between teachers’ instructions and students’ monitoring accuracy. Instead of relying on self-report measures, the authors used classroom observations to assess teachers’ SRL practice and learning judgement to measure students’ monitoring. The findings of this research accent the importance of child-centered instruction. The authors emphasize that not only the content of instruction but also the type of instruction plays an important role for supporting SRL in the classroom.

Two commentaries on the special issue are also provided. The first is by Deborah Butler, whose work on strategic and self-regulated learning (e.g., Butler, 1998; Butler, 2015), and in particular on the collaboration and co-regulation in teachers’ professional development (e.g., Butler et al., 2004; Butler & Schnellert, 2012) has functioned as a guide for many of the authors in this special issue. The second is a commentary by Jeff Greene, who has examined how people can develop their capacity to self-regulate their learning in numerous studies (e.g., Schunk & Greene, 2018) by applying both experimental and non-experimental designs as well as quantitative, qualitative and mixed methods (e.g., Greene & Azevedo, 2009; 2010).

We believe that the articles in this special issue and the following reflections present a critical analysis of teachers’ perceptions and practices for supporting SRL in the classroom and provide a significant foundation for future research.

We would like to thank all the authors and reviewers for their contribution to this special issue. Special thanks to Roger Azevedo, the previous Editor of Metacognition and Learning Journal, for his support in starting this special issue, and to Anastasia Efklides, the current Editor of Metacognition and Learning, for supervising this special issue until now. Many thanks to the two commentators, Deborah Butler and Jeff Greene, for their thoughtful and inspiring reflections on this collection of papers.
